# Daily intake of antioxidants ameliorates PM_2.5_-induced neuronal injury in mice

**DOI:** 10.3389/fnut.2026.1806418

**Published:** 2026-03-31

**Authors:** Taida Huang, Hui Li, Xiaomin Huang, Bo Li, Hongyi Zhang, Xu Bai, Brian G. Oliver, Meilin Tian, Chenju Yi, Dan Li, Hui Chen

**Affiliations:** 1 Department of Geriatrics, Seventh Affiliated Hospital of Sun Yat-sen University, Shenzhen, China; 2 School of Life Sciences, Faculty of Science, University of Technology Sydney, Ultimo, NSW, Australia; 3 Respiratory Cellular and Molecular Biology, Woolcock Institute of Medical Research, Macquarie University, Macquarie, NSW, Australia; 4 Shenzhen Institutes of Advanced Technology, Chinese Academy of Sciences, Shenzhen, China; 5 Guangdong Provincial Key Laboratory of Digestive Cancer Research, The Seventh Affiliated Hospital of Sun Yat-sen University, Shenzhen, China; 6 Shenzhen Key Laboratory of Chinese Medicine Active Substance Screening and Translational Research, Shenzhen, China; 7 Department of Pathology, The First Affiliated Hospital of Gannan Medical University, Ganzhou, Jiangxi, China

**Keywords:** mitochondria, NAC, neurotoxicity, oxidative stress, vitamin C

## Abstract

**Objective:**

Fine particulate matter (PM_2.5_) within polluted air is a significant health risk and a strong oxidant. Even exposure to low-level PM_2.5_ (at or below the WHO standard) is linked to neuroinflammatory, oxidative stress and the development of neurological disorders in the long term. This study aimed to investigate the prophylactic effects of antioxidant supplementation on mitigating sub-chronic exposure to low-level PM_2.5_-induced brain pathology in mice.

**Materials and methods:**

Mice were exposed to traffic-derived PM_2.5_ (5 μg/day) collected from a highway in Sydney, Australia, once daily for 3 weeks, while receiving antioxidant, vitamin C (10 mM) or N-acetylcysteine (NAC, 40 mM), via drinking water. Oxidative stress and neuronal loss were assessed across different brain regions. *In vitro* experiments were conducted to evaluate neuronal integrity and mitochondrial function by VC and NAC treatment in PM_2.5_-exposed primary neurons.

**Results:**

Sub-chronic PM_2.5_ exposure increased lipid peroxidation and reduced neurofilament density in the cortex, hippocampus, and thalamus, which were mitigated by VC and NAC supplementation. Although oxidative stress (ROS accumulation and increase in 4-HNE) was prevented in all brain regions by VC and NAC, neurofilament loss remained. *In vitro*, VC and NAC reduced mitochondrial ROS production, which in turn improved neuronal and synaptic survival, suggesting mitochondria-dependent oxidative stress plays a central role in PM_2.5_-induced neurotoxicity at least in the cortex and hippocampus.

**Conclusion:**

Mitochondria-dependent oxidative stress is a key mechanism underlying PM_2.5_-induced neurotoxicity, which can be attenuated by VC and NAC supplementation. This highlights a potential prophylactic strategy to partially protect the brain from polluted air.

## Graphical abstract

Schematic graphic abstract. Sub-chronic PM2.5 exposure induces mitochondria-derived oxidative stress, leading to the loss of neurofilaments and synapses of neurons. Daily intake of antioxidants such as VC or NAC attenuates this PM2.5-induced neurotoxicity and improves mitochondrial function by reducing ROS levels in mouse neurons.
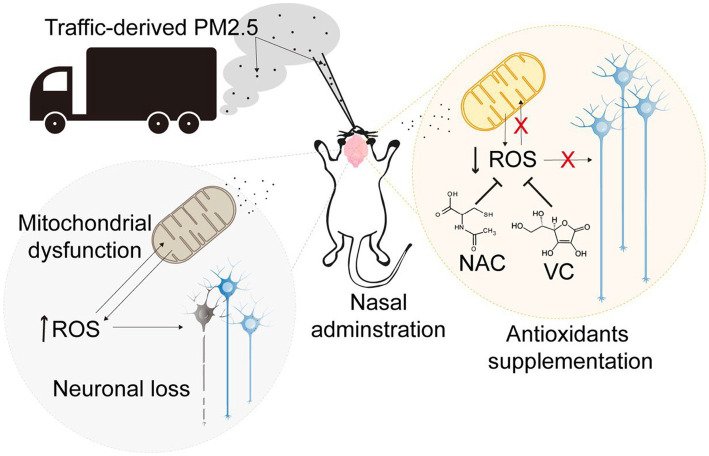

## 1 Introduction

Air pollution remains a critical global public health challenge. Among the various airborne contaminants, fine particulate matter with a diameter less than 2.5 μm (PM_2.5_) is a key health threat, due to its ability to traverse the lung’s epithelial-endothelial barrier, entering the bloodstream via the alveoli, causing systemic health impacts (1). A growing body of evidence indicates that PM_2.5_ can further cross the blood–brain barrier, provoking neuroinflammation and oxidative stress in the brain (2, 3). Furthermore, the olfactory route is another potential entry route of PM_2.5_ into the brain (4, 5). This neurotoxicity of PM_2.5_ has been linked to multiple neurological disorders, including cognitive impairment and elevated risk of neurodegenerative diseases, such as Alzheimer’s disease (AD), Parkinson’s disease (PD), amyotrophic lateral sclerosis (ALS), as well as stroke and depression (6–9).

PM_2.5_ is a potent oxidant due to the surface attached metals and organic compounds. Upon inhalation, they can disrupt redox balance and result in systemic oxidative stress (10–13). Oxidative stress occurs when endogenous antioxidants are overwhelmed, typically inducing mitochondrial injury. This, in turn, impairs ATP production that is critical to support cellular functions, thereby increasing apoptosis (14–18). Even PM_2.5_ levels that have been considered “safe,” such as in countries like Australia, can still cause neuronal damage. Indeed, previously, we showed that *in utero* exposure to such a low-level of PM_2.5_ induced hippocampal neuronal loss, synaptic impairment, and cognitive impairment in adulthood, associated with the overproduction of mitochondrial reactive oxygen species (ROS), disruption in mitochondrial electron transport chain elements, and mitochondrial dysfunction (17). These findings suggest that there is no safe level of PM_2.5_. While stricter air quality regulations and advancing technology to reduce ambient levels of harmful PM_2.5_ may take decades to achieve, effective strategies are needed to mitigate the adverse effects on brain health.

PM_2.5_ may initiate Fenton reactions, generating hydroxyl radicals that damage the mitochondrial electron transport chain to impair ATP synthesis and promote programmed cell death (19, 20). Given the central role of oxidative stress in PM_2.5_-induced neurotoxicity, antioxidants naturally represent a preferred therapeutic strategy. Over-the-counter antioxidants, such as Vitamin C (VC) and N-acetylcysteine (NAC), have demonstrated protective effects against various oxidative insults, such as tobacco smoking, neuroinflammation, and aging, including rescuing declined cognitive functions (21–27). While the efficiency of antioxidants in the clinic has always been questioned, the lack of positive findings in those published trials can often be limited by the choice of antioxidant, and many adopted low doses with limited therapeutic effects (28). Nevertheless, whether antioxidants can ameliorate or prevent PM_2.5_-exposure induced neurological changes remains unclear, which formed the rationale of this study.

In this study, we sub-chronically exposed mice to low-level PM_2.5_ using our well-established protocol that was shown to cause neuronal pathology (17), with prophylactic VC or NAC treatment. *In vitro* studies using primary mouse neurons were used to investigate the impact on mitochondrial function and neuronal integrity. We found that PM_2.5_ induced neuronal pathology was through mitochondria-dependent oxidative stress, and that both VC and NAC supplements attenuated the detrimental effects of PM_2.5_ on neural axon integrity and synaptic plasticity in the cortex and hippocampus by suppressing oxidative stress and apoptosis.

## 2 Materials and methods

### 2.1 PM_2.5_ collection

PM_2.5_ were collected as previously described (29). Briefly, traffic-derived PM_2.5_ were collected from a highway in Sydney, New South Wales, Australia, in June 2022 using Smart Particulate Flow Samplers configured for total suspended particles and PM_2.5_ (8 L/min flow rate), with 47 mm Teflon filters (Pall Life Sciences, USA). PM_2.5_ were extracted from the filters using 90% ethanol with 5 min of sonication, followed by freeze drying. PM_2.5_ was then suspended in saline stored at −20 °C before the experiments as previously described (17). The heavy metal composition was published in the study on lung health on the same mice (30).

### 2.2 Animal experiments

All animal procedures were approved by the Animal Care and Ethics Committee at the University of Technology Sydney (approval# ETH19-4303) and performed following the Australian National Health and Medical Research Council’s Guide for the Care and Use of Laboratory Animals. To minimise the variability introduced by sex hormone fluctuation, male BALB/c mice (6 weeks old, Animal Resources Centre, WA, Australia) were used to prove the concept by giving daily intranasal instillations of either PM_2.5_ (5 μg/day; PM_2.5_ group) or saline (CTL group) for 3 weeks (13, 17). This PM_2.5_ level represents low-level air pollution based on the dose-conversion published in our previous paper (17). In subgroups of PM_2.5_-exposed mice, VC (10 mM) or NAC (40 mM) were supplied in the drinking water at the same time when PM_2.5_ exposure was initiated. These doses were chosen based on previous studies on the protective effects of VC and NAC against cigarette smoke-induced lung pathology, which is the first entry of PM_2.5_ into the body (31, 32). Thus, male BALB/c mice were randomly assigned to 4 experimental groups, including CTL, PM_2.5_, PM_2.5_ + VC and PM_2.5_ + NAC. All animal handling and treatments were performed within the same time window each day and by the same operator to minimise potential confounders related to time-of-day effects and inter-operator variability. At the end point, mice were deeply anesthetised by isoflurane (2%–3%), followed by decapitation. Brains were quickly removed and fixed in 4% paraformaldehyde at 4 °C overnight, followed by dehydration and paraffin embedding.

### 2.3 Immunofluorescent staining

Paraffin-embedded mouse brain tissues were coronally sectioned into 5 μm slices by a microtome (Leica, Germany). After deparaffinisation and rehydration through xylene and graded ethanol, sequentially, antigen retrieval was performed by soaking slides in citrate buffer (pH = 6.0, Servicebio, China) at 100 °C for 15 min. The immunofluorescence staining procedures were performed using our previously published methods (33).

Sections were incubated with primary antibodies at 4 °C overnight, followed by the corresponding fluorophore-conjugated secondary antibodies at the manufacturer’s recommended dilutions for 1 h at 4 °C. All primary antibodies used in this study are routinely utilised in our laboratory and have been extensively validated for mouse brain IHC in our previous work. For quality assurance, each new antibody aliquot is cross-validated with previous batches. All antibodies were commercially validated with verified mouse species reactivity, as documented by the manufacturers’ datasheets. Negative controls, performed by omitting the primary antibody, showed no non-specific staining. Specifically, the following antibodies were used: rabbit anti-Neuronal Nuclei (NeuN, 1:500, ab177487, Abcam, USA), rabbit anti-Neurofilament 200 kDa (NF200, 1:500, N4142, Merck, USA), mouse anti-Superoxide Dismutase 1 (SOD1, 1:300, 4266P CST, USA), mouse anti-4-Hydroxynonenal (4-HNE, 1:200, NB100-63093, NOVUS, USA), mouse anti-Beta III Tubulin (Tuj1, 1:500, 801201, Biolegend, USA), rabbit anti-Synapsin I (SYN1, 1:1000, 5,297, CST, USA), rabbit anti-Cleaved Caspase-3 (Cleaved-CASP3, 1:500, 9661, CST, USA), Rabbit anti-Manganese Superoxide Dismutase (MnSOD, 1:300, Merck, USA). Images were acquired using a spinning disk confocal microscope (SpinSR, Olympus, Japan) or a slide scanner (VS200, Olympus, Japan).

### 2.4 MitoSOX staining and seahorse assay in PM_2.5_ treated primary mouse neurons *in vitro*

Primary cortical neurons were isolated from the 17.5 day-old (E17.5) embryos with BALB/c background. 3–4 brains were used per 24-well culture plate with a seeding density of 1 × 10^6^ cells/well. After 3 days of culture in Neurobasal medium (21103049, Gibco, USA) containing B27 supplement (Gibco, USA), neurons were exposed to PM_2.5_ (50 μg/mL) for 16 h using our previously published protocol (17). Simultaneously, in PM_2.5_-exposed neurons, 200 μM VC (34) or 1 mM NAC (35) were added to different subgroups for 16 h. Mitochondrial function was assessed by Seahorse XF Pro Analyser and Mito Stress Test kit (103015–100, Agilent, USA) according to the manufacturer’s protocol. Oligomycin (Oligo, 1.5 μM) was used to inhibit ATP synthase. Carbonyl cyanide-p-trifluoromethoxy phenylhydrazone (FCCP, 2 μM) was used to maximise electron flux to reflect the maximal respiratory capacity of mitochondria. Rotenone (Rot, 0.5 μM) and Antimycin A (AA, 0.5 μM) were added at the end of the experiment for non-mitochondrial oxygen consumption. Mitochondrial respiration was measured by oxygen consumption rate (OCR) which is normalised to the cell number in each well indicated by nuclear staining with Hoechst after testing.

### 2.5 TUNEL assay in PM_2.5_ treated primary mouse neurons *in vitro*

Apoptosis-associated DNA fragmentation was assessed by TUNEL using a commercial kit according to the manufacturer’s protocol (T2194, Solarbio, China). Live-cell mitochondrial superoxide was measured using MitoSOX reagent (M36008, ThermoFisher, USA) following the manufacturer’s instructions.

### 2.6 Statistical methods

The sample size was determined based on a prior pilot study and 3R principles to minimise animal use. For staining analysis, a minimum of three non-adjacent sections per mouse were selected for each staining protocol, encompassing the entire cortical, hippocampal, and thalamic regions. For each section, five randomly chosen, non-overlapping visual fields at ×20 magnification were acquired and subjected to quantitative analysis using ImageJ software (National Institutes of Health, USA). Outcome assessment was performed by an investigator blinded to group allocation, and data analysis was conducted using coded group labels until analyses were completed. Relative fluorescence intensity was normalised to the mean intensity of the control (CTL) group. Results are presented as mean ± SEM. For all analyses, *n*-values represent the number of mice per group, which are reported in the figure legends. Data distribution was tested to be normal using the Shapiro–Wilk test, and the differences between groups were analysed by one-way ANOVA followed by Tukey’s *post hoc* tests for multiple-comparison using Prism 9.0 (GraphPad Software, USA). *p* < 0.05 was considered statistically significant.

## 3 Results

### 3.1 Antioxidant supplements mitigated neurofilament loss caused by PM_2.5_ exposure in mice

**Figure 1:**
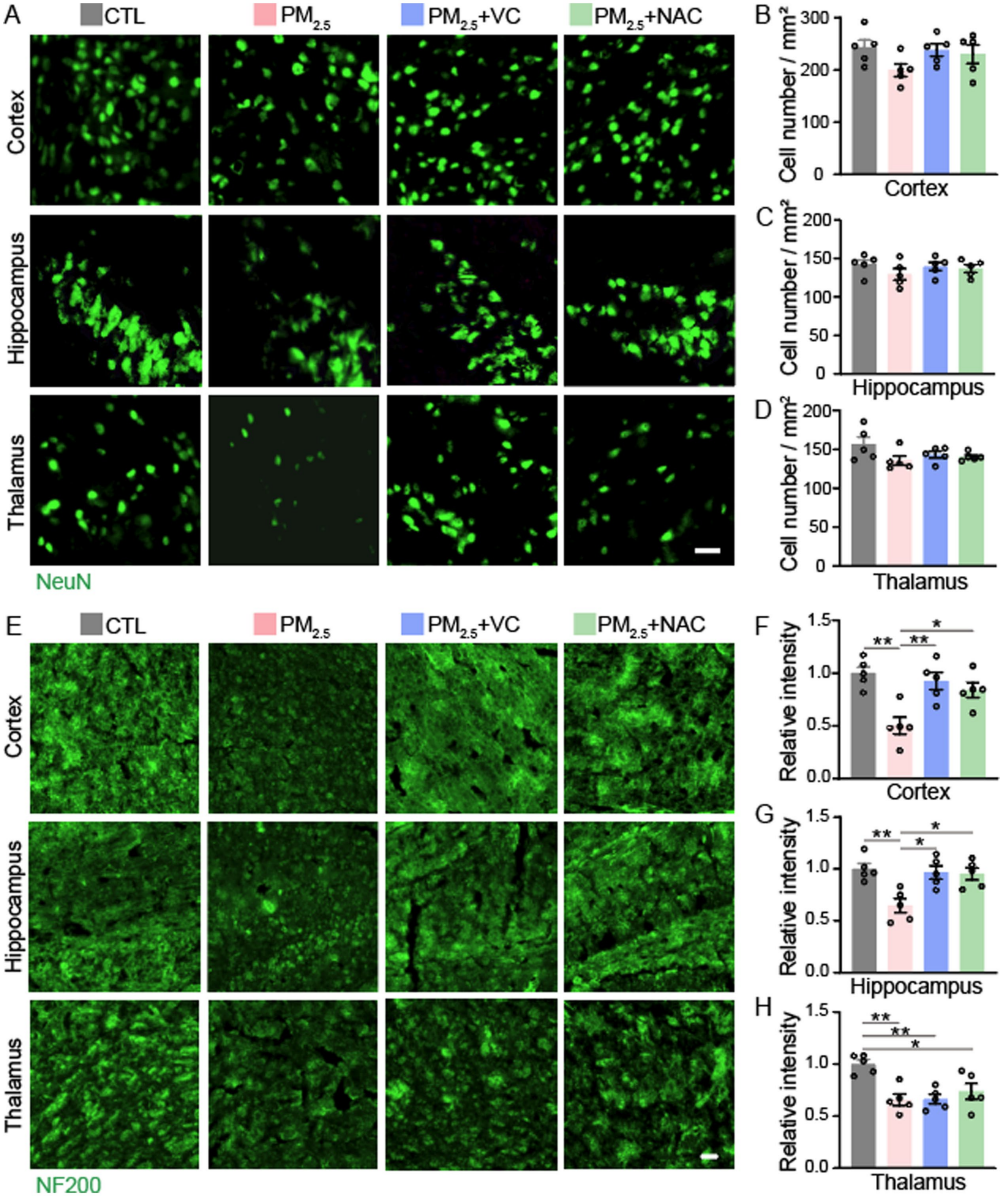
Immunofluorescent staining of neuronal markers in mouse brains. Representative images of NeuN staining **(A)** and quantification in the cerebral cortex **(B)**, hippocampus **(C)**, and thalamus **(D)**. Representative images of NF200 staining **(E)** and quantification normalised to the control (CTL) group in the cortex **(F)**, hippocampus **(G)**, and thalamus **(H)**. Results are presented as mean ± SEM; *n* = 5. Scale bar = 20 μm. *p < 0.05, **p < 0.01.

To evaluate brain neuronal pathology induced by PM_2.5_ exposure in mice, we performed immunofluorescence staining for NeuN and NF200 for neuronal nuclei and neurofilaments, respectively. PM_2.5_ induced 19.6% and 17.4% reduction in neuron density in the cortex and hippocampus, although no significant difference was observed compared to the control group (Figures 1A–D). However, neurofilament density was significantly reduced in the cortex, hippocampus, and thalamus of PM_2.5_-exposed mice (Figures 1E–H).

In PM_2.5_-exposed mice, daily supplementation with VC and NAC in drinking water significantly alleviated neurofilament loss in the cortex and hippocampus (Figures 1F,G), but not in the thalamus (Figure 1H).

### 3.2 Antioxidant supplements suppressed brain oxidative stress in mice

**Figure 2:**
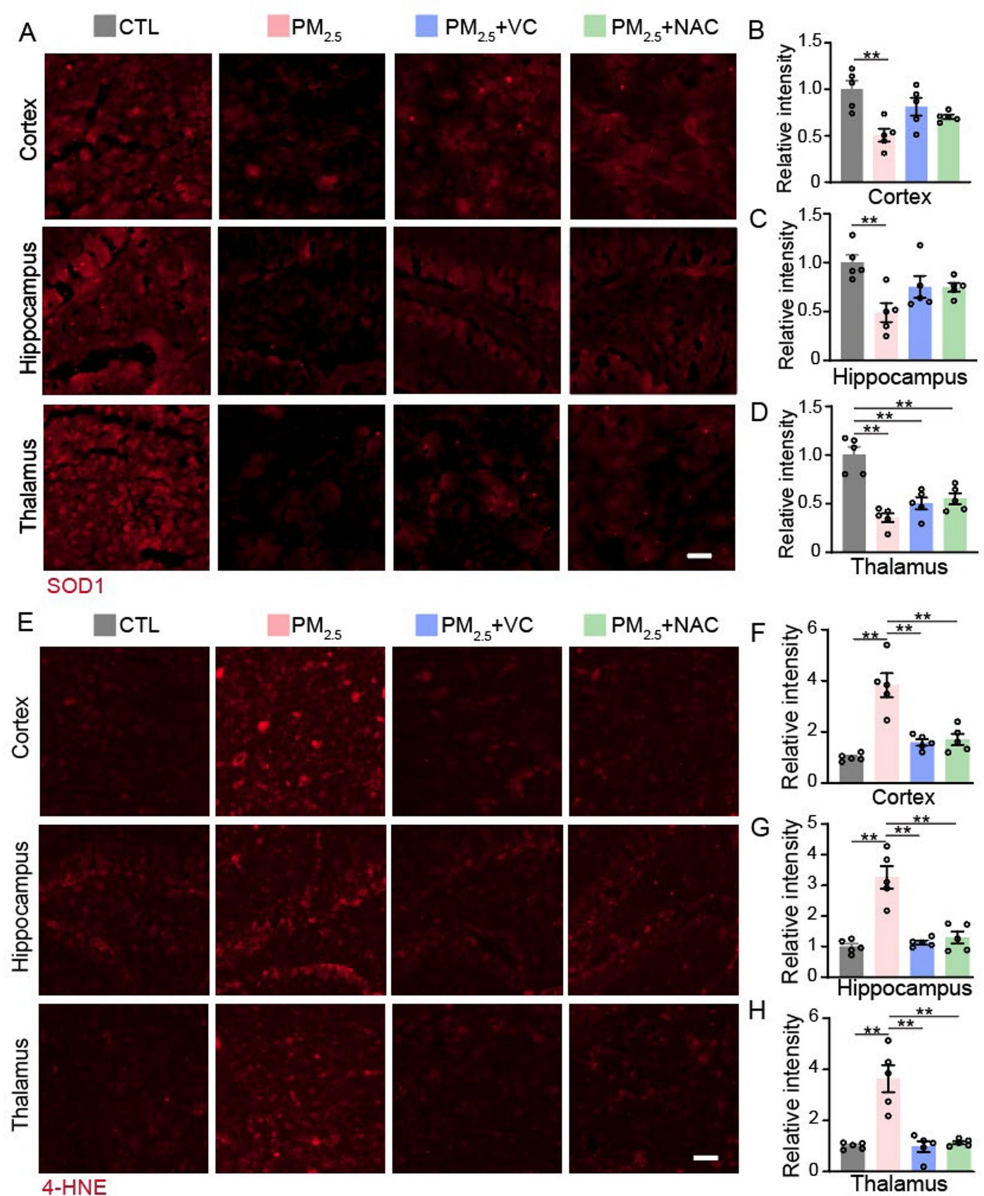
Immunofluorescent staining of oxidative stress markers in mouse brain sections. Representative images **(A)** and quantification of SOD1 staining in cortex **(B)**, hippocampus **(C)**, and thalamus **(D)**. Representative images **(E)** and quantification of 4-HNE staining in the cortex **(F)**, hippocampus **(G)**, and thalamus **(H)**. Results are presented as mean ± SEM; *n* = 5. Scale bar = 20 μm. *p < 0.05, **p < 0.01.

PM_2.5_ are potent oxidants. To assess the impact of PM_2.5_ exposure and antioxidant supplements on oxidative stress injury in the brain, the levels of endogenous antioxidative enzyme SOD1 (Figure 2A) and 4-HNE (Figure 2E) reflecting lipid peroxidation were measured. PM_2.5_ exposure significantly reduced the SOD1 protein and increased 4-HNE levels in all 3 regions measured here, including cortex (Figures 2B,F), hippocampus (Figures 2C,G), and thalamus (Figures 2D,H). Daily supplementation with VC or NAC increased SOD1, albeit without statistical significance (Figures 2B–D), and both nearly normalised the 4-HNE levels, respectively (Figures 2F–H).

### 3.3 VC and NAC enhanced neurite growth and synaptic formation of PM_2.5_ treated neuronal cells *in vitro*

**Figure 3:**
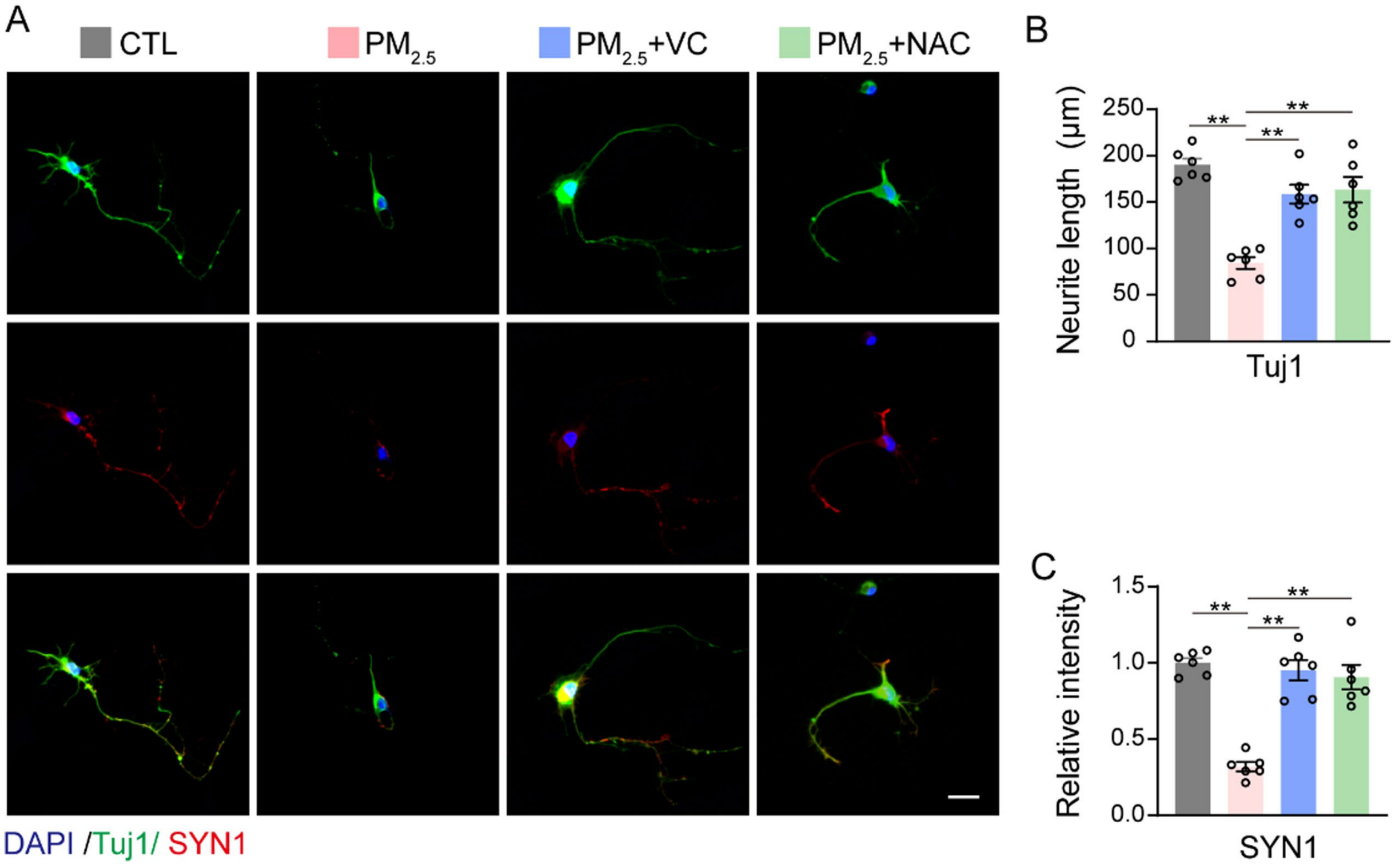
VC and NAC mitigated PM_2.5_-induced neurite and synaptic impairment. Representative images **(A)** and quantification of Tuj1 [pan-neurite marker, **(B)**] and SYN [pre-synaptic marker, **(C)**] in neuronal cells. Results are presented as mean ± SEM; *n* = 5. Scale bar = 20 μm. *p* < 0.01.

To further evaluate the neuroprotective effects of VC and NAC, Tuj1 and SYN1 staining were performed in primary cortical neurons to determine the neurite growth and synaptic plasticity, respectively (Figure 3A). PM_2.5_ exposure significantly reduced neurite length (Figure 3B) and SYN1 expression compared with those in the CTL groups (Figure 3). The addition of VC or NAC to the culture medium significantly increased neurite length in PM_2.5_-exposed neurons (Figure 3B), while normalising SYN1 levels (Figure 3C). These results indicated that both VC and NAC are capable of protecting neurites and synaptic plasticity in neurons following PM_2.5_ exposure *in vitro*.

### 3.4 VC and NAC reduced neuronal cell death caused by PM_2.5_ *in vitro*

**Figure 4:**
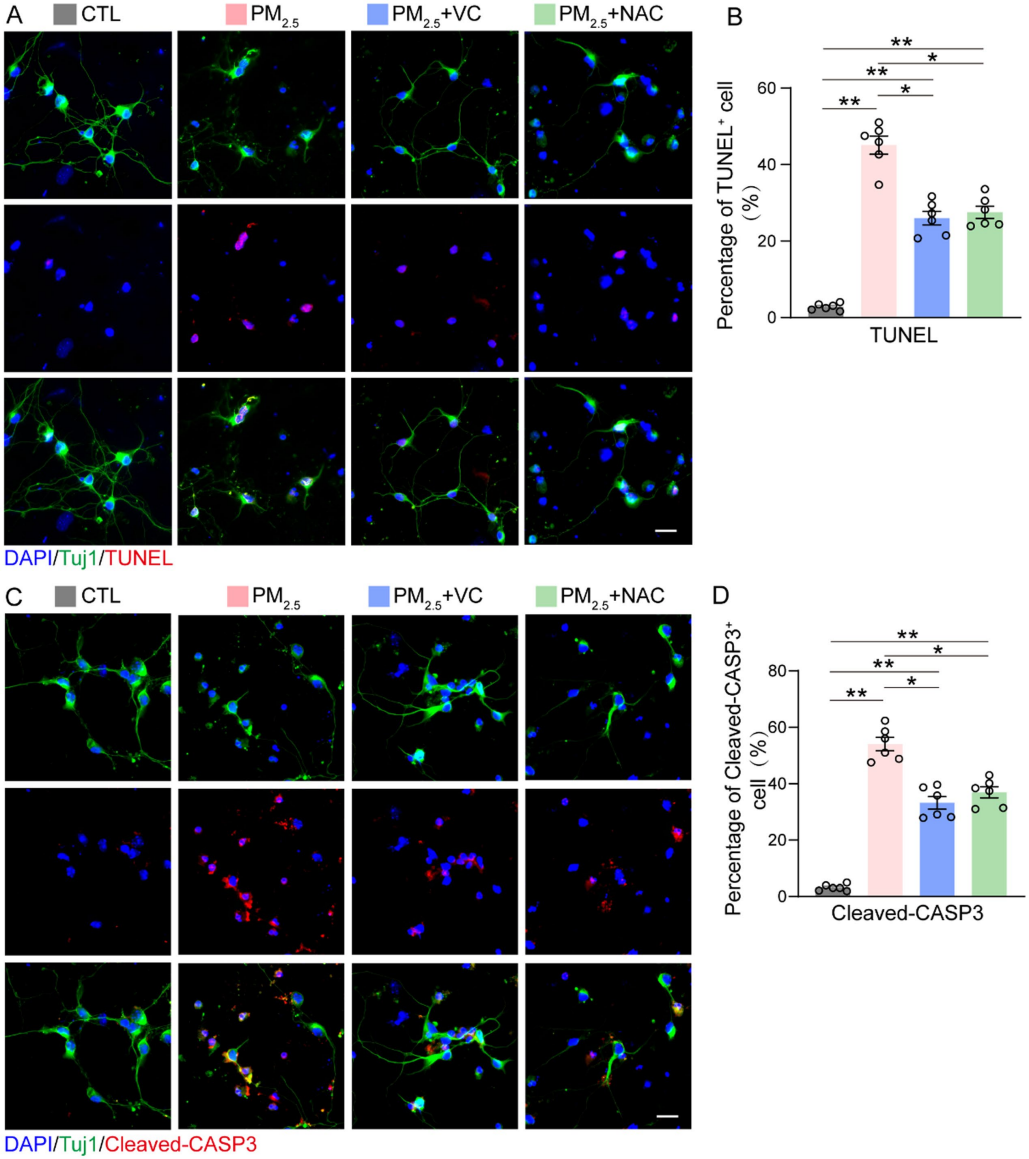
VC and NAC ameliorated PM_2.5_-induced neuronal cell death *in vitro*. Representative images **(A)** and quantification **(B)** of TUNEL and Tuj1 co-immunostaining (TUNEL^+^ and Tuj1^+^/Tuj1^+^) in neurons. Representative images **(C)** and quantification **(D)** of cleaved caspase-3 (Cleaved-CASP3; apoptosis marker) and Tuj1 co-staining (Cleaved-CASP3^+^ and Tuj1^+^/Tuj1^+^) in neurons. Results are presented as mean ± SEM; *n* = 5. Scale bar = 20 μm. *p < 0.05, **p < 0.01.

The neuronal cell death caused by PM_2.5_ exposure was evaluated using a TUNEL assay, which reflects genomic DNA breaking during apoptosis (Figure 4A) and immunofluorescent staining of Cleaved-Caspase 3 (Cleaved-CASP3), which is the active form of Caspase 3 involved in apoptosis (Figure 4C). PM_2.5_ exposure resulted in a marked increase in the percentages of both TUNEL-positive (Figure 4B) and Cleaved-CASP3-positive (Figure 4D) neuronal cells. The addition of VC or NAC mitigated the increase in apoptotic markers in PM_2.5_-exposed neurons (Figures 4B,D).

### 3.5 VC and NAC reduced the mitochondrial superoxide and improved the respiratory function in PM_2.5_ treated neuronal cells *in vitro*

**Figure 5:**
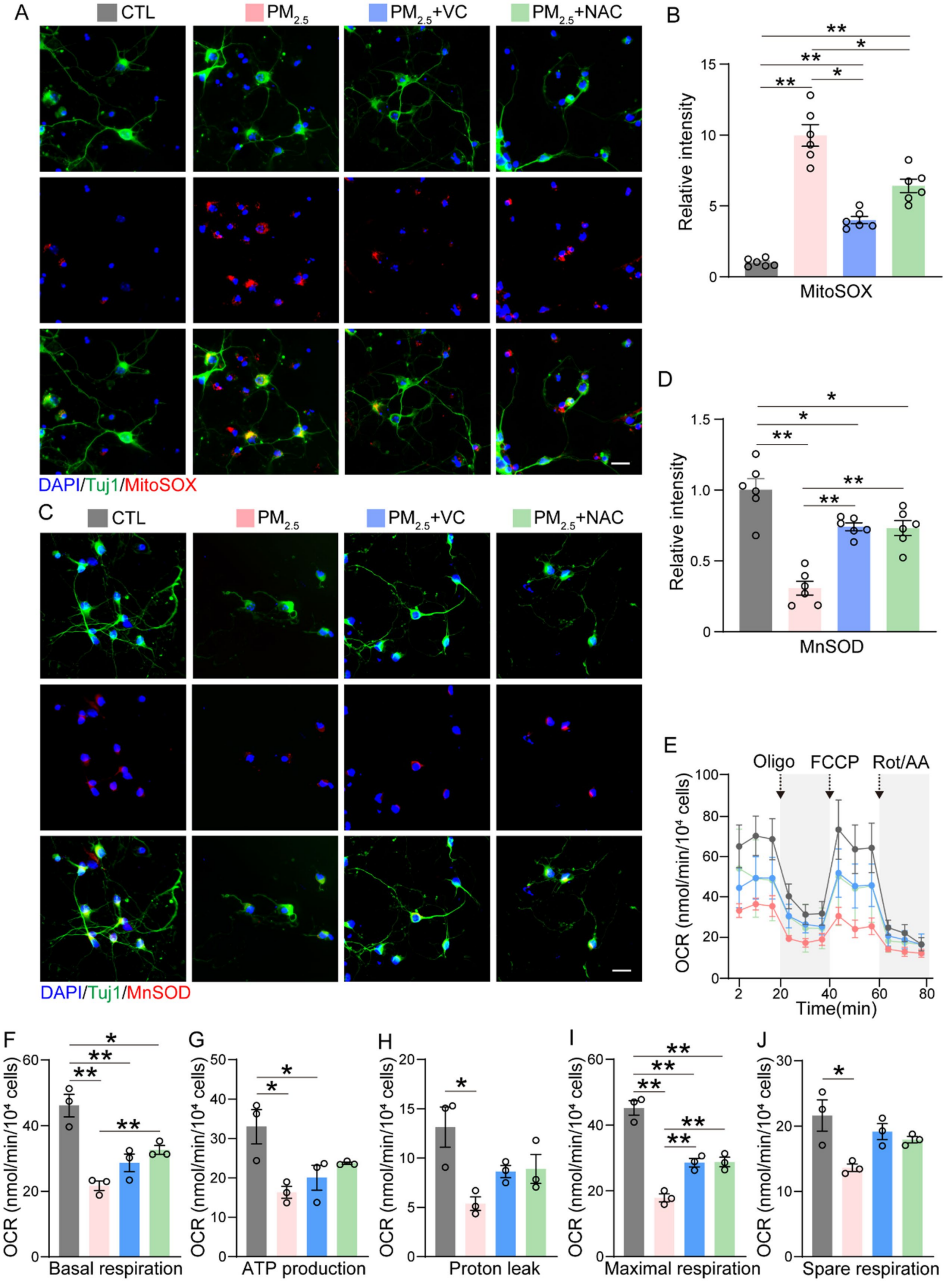
VC and NAC reduced mitochondrial superoxide in PM_2.5_-exposed neuronal cells *in vitro*. Representative images **(A)** and quantification **(B)** of Tuj1 and MitoSOX (mitochondrial superoxide probe) co-staining. Representative images **(C)** and quantification **(D)** of Tuj1 and MnSOD (mitochondrial antioxidant) co-staining. Mitochondrial respiratory function in PM_2.5_-exposed primary neurons treated with VC or NAC by seahorse assay **(E–J)**. Results are presented as mean ± SEM; *n* = 5 in **(B,D)**; *n* = 3 in **(E–J)**. Scale bar = 20 μm. *p < 0.05, **p < 0.01.

Mitochondria are vulnerable to oxidative stress, as shown in our previous study (17). PM_2.5_ significantly increased oxidative stress marker MitoSOX and reduced antioxidant MnSOD in neurons (Figure 5). Both VC and NAC suppressed MitoSOX levels in PM_2.5_-exposed neurons, more effectively in the VC group (Figures 5A,B). MnSOD levels were increased twofold in the VC and NAC treated groups, respectively (Figures 5C,D).

The mitochondrial function, including respiratory activity at baseline and under stress, was determined by Seahorse assay (Figure 5E), in which PM_2.5_ significantly reduced basal respiration, ATP production, proton leak and maximal respiration of primary neurons (Figures 5F–J). VC significantly increased the maximal respiration (Figure 5I) in PM_2.5_-exposed neurons, although no significant difference was observed in basal respiration, ATP production, or proton leak (Figures 5F–H). NAC significantly increased the basal and maximum respiration (Figures 5F,I), while no statistically significant changes were observed in ATP production and proton leak in PM_2.5_-exposed neurons (Figures 5G,H).

## 4 Discussion

This study provides robust evidence that the early stage neuronal injury due to sub-chronic exposure to low-level PM_2.5_ (even within the range considered “safe”) is associated with a marked reduction in neurofilament density across multiple brain regions, a key early-stage structural compromise without significantly affecting neuronal cell count. This is likely driven by increased apoptosis and mitochondrial dysfunction. Importantly, daily supplementation with antioxidants, VC and NAC, significantly mitigated these neurotoxic effects by reducing oxidative stress and related mitochondrial dysfunction, lipid damage, and apoptosis, which in turn improved neural axonal integrity and synaptic plasticity. Taken together, our results highlight mitochondria-dependent oxidative stress as a central mechanism of PM_2.5_-induced neuronal injury and support the prophylactic effect of antioxidant supplementation by partially preventing air pollution–exposure induced neuronal damage in key brain regions (see Graphical abstract).

The most significant finding of this study is that oxidative stress is a key driver of PM_2.5_-induced neurofilament degradation at the early stage, which may be the starting point of impaired synaptic plasticity and neurocognitive decline in the long term, while increased risk of dementia is common among individuals living in polluted environments (2, 36). As the primary source of cellular ROS, mitochondria also possess a sophisticated antioxidant system to scavenge excess ROS (37). This process is expected to effectively counteract any increase in the cues of oxidative stress, such as cellular ROS levels. Here, we observed how this endogenous protective mechanism responded adaptively at the early stage of PM_2.5_ exposure; however, it failed to protect against PM_2.5_-induced oxidative damage in the brain. This early-stage event *in vivo* was further replicated in the primary neurons *in vitro*. Our findings of increased endogenous antioxidant levels associated with increased oxidative stress, impaired mitochondrial function, and related injuries to cellular lipid components were different from later-stage findings, where endogenous antioxidants are exhausted due to prolonged oxidative stress (38). However, we are unable to exclude the possibility of misfolding SOD1, commonly seen in neurodegenerative diseases (39), which can still be stained using commercial antibodies. Future studies can address the subtypes of SOD1 in this setting. Nevertheless, our findings align with previous reports linking PM_2.5_ exposure to increased oxidative stress (17, 37).

Our interventional results extend the role of oxidative stress to the specific mitochondrial involvements under both neuropathology and therapeutic targets for neuroprotection, as shown by the results of VC and NAC treated mice and primary neuron cells. The overall benefits for neurocognition of both antioxidants have been shown in the literature (40, 41); however, the evidence of such benefits in the context of PM_2.5_ exposure, especially *in vivo*, is scanty (42). VC is a water-soluble vitamin that directly scavenges ROS, such as superoxide, hydroxyl radicals, and peroxyl radicals; while NAC mainly acts as a *precursor* for glutathione, which is a major endogenous antioxidant in addition to SOD1, and plays a minor role as a direct scavenger of ROS. However, VC seems more potent than NAC in increasing systemic antioxidant capacity (43, 44). Indeed, the addition of either VC or NAC normalised the response of SOD1 to PM_2.5_ exposure, with no significant difference observed in the levels of lipid peroxide marker 4-NHE and neurofilament in two key brain regions involved in neurocognition (cortex and hippocampus). However, NAC seems to exert better mitochondrial protection than VC, which may be attributed to its capacity to replenish glutathione. These results suggest a well-restored antioxidant capacity against additional oxidative stress due to environmental exposure, which is key to preserving neuronal integrity, particularly synaptic loss. This is highly relevant in reducing the risk of dementia imposed by PM_2.5_ exposure, given that synaptic loss and impaired plasticity are early hallmarks of neurodegenerative diseases such as AD (45). Notably, a previous study suggested a resulting serum VC concentration of ~ 5.5–6 μg/mL (31), and the NAC body content of 1 g/kg of body weight (32) with the doses used in this study.

The interplay between PM_2.5_-induced oxidative stress and cellular metabolic regulation is complex. Emerging evidence indicates that mammalian target of rapamycin (mTOR) signalling, a central regulator of cellular energy sensing and nutrient metabolism, is highly sensitive to environmental toxicants (46). mTOR complex 1 (mTORC1) activation relies on upstream signalling processes, including Rab1A-mediated signal relay, and mTORC1-dependent phosphorylation events are critical for maintaining protein stability and cellular functional integrity (47). PM_2.5_-induced oxidative stress may disrupt these upstream sensing mechanisms, resulting in impaired mTORC1 recruitment and activation of downstream signalling pathway elements (48). Moreover, PM_2.5_-associated mitochondrial dysfunction and redox imbalance share mechanistic features with biological ageing, a process characterised by dysregulation of metabolic hubs, such as mTOR and its upstream regulator Peroxisome proliferator-activated receptor coactivator 1α, that may progress neural degeneration (49). In this context, our observation that VC and NAC mitigate mitochondrial impairment induced by PM_2.5_ exposure suggests that these antioxidants may indirectly preserve mTOR-related metabolic homeostasis, although direct effects on mTOR signaling were not assessed and warrant further investigation.

However, we need to acknowledge the lack of protection in the thalamus by either antioxidant. The partially mitigated cell apoptosis markers in the *in vitro* study may help to explain such incomplete protection. It has been suggested that glutathione can increase the stability of VC *in vivo*, while the synergistic beneficial role of VC and NAC has also been suggested based on their chemical properties and biological effects (43, 44). Future studies can evaluate the combination of VC and NAC to investigate whether such an approach can exert complete protection of the brain against PM_2.5_ exposure induced neuropathology. While our in vitro data strongly suggest PM_2.5_-induced mitochondrial functional impairment can be ameliorated by the antioxidants, the definitive causal linkage in the *in vivo* setting requires further investigation using *in situ* tissue mitochondrial function analysis. Another limitation is the short duration of this study. In our unpublished cohort, a minimum of 4 weeks is needed to cause significant cognitive changes. Therefore, the 3-week exposure paradigm in this study represents an early-stage neuropathology, and future studies can extend the duration to examine the impact on neurocognition in the long term, such as learning and memory functions, to correlate histological findings with functional outcomes. The absence of dose-response analyses for antioxidant treatments can also be addressed in the future with reference levels used in this study. It needs to be noted that intranasal installation was used to model PM_2.5_ inhalation in humans. Modeling human inhalation in mice is relatively difficult; we chose intranasal instillation as we could accurately deliver a fixed dose to the lung, whereas in spontaneous breathing this would be more difficult and probably requires continuous exposure. There are also differences in where particles deposit in the lung between mice and humans. PM_2.5_ and smaller fine particles exhibit greater penetration into the distal lung, including the bronchioles and alveoli, where sedimentation and diffusion are the primary deposition mechanisms. These principles are conserved across species, but their relative contributions differ in mice due to higher respiratory rates, shorter airway lengths, and a distinct branching architecture compared with humans. As a result, mice generally experience relatively higher distal lung deposition of fine particles for a given inhaled mass. The latest WHO guideline recommends “annual average of PM_2.5_ ≤ 5 μg/m^3^ and a 24 hour average of ≤15 μg/m^3^.” As such, our model represents low-level polluted air, which may not be applied to those living in areas with much higher PM_2.5_ levels. Future studies can adopt higher PM_2.5_ dose regimes to validate the protective effects of antioxidants to enable broader translation. In addition, only males were studied here. We prioritised this sex based on the human observation of high susceptibility to PM_2.5_ induced brain pathology and adverse cognitive outcomes in young males, while females seem more vulnerable at older ages after menopause (50). However, females still show white matter changes using brain imaging analysis (50). Although this does not necessarily correlate to any functional changes, it is unclear about its long-term impact. Therefore, future studies can consider a longitudinal study design to determine if antioxidant supplements can mitigate or reverse the imaging abnormalities and cognitive performance, especially after menopause, in females.

In conclusion, our findings reinforce the notion that mitochondria-dependent oxidative stress is a key mechanism underlying PM_2.5_-induced neurite and synaptic injury ahead of neuronal loss due to increased apoptosis. Daily supplementation with VC or NAC offers a promising strategy to mitigate these effects. These results provide a potential prophylactic approach to safeguard brain health in polluted environments (see Graphical abstract). Future research can aim at translating this strategy in high-risk populations.

## Data Availability

The original contributions presented in the study are included in the article/supplementary material, further inquiries can be directed to the corresponding authors.
